# GeneXpert on patients with human immunodeficiency virus and smear-negative pulmonary tuberculosis

**DOI:** 10.1371/journal.pone.0253961

**Published:** 2021-07-06

**Authors:** Nguyen Kim Cuong, Nguyen Bao Ngoc, Nguyen Binh Hoa, Vu Quoc Dat, Nguyen Viet Nhung

**Affiliations:** 1 Department of Tuberculosis and Lung Disease, Hanoi Medical University, Hanoi, Vietnam; 2 Department of Respiratory Tuberculosis, National Lung Hospital, Hanoi, Vietnam; 3 Department of Pharmacy, National Lung Hospital, Hanoi, Vietnam; 4 National Tuberculosis Programme, Hanoi, Vietnam; 5 Department of Infectious Diseases, Hanoi Medical University, Hanoi, Vietnam; Institute of Medical Sciences, Banaras Hindu University, INDIA

## Abstract

**Objectives:**

Vietnam is a high-prevalence country for tuberculosis (TB). Xpert MTB/RIF is a novel PCR-based diagnostic test that is substantially more sensitive for detecting *M*. *tuberculosis* than traditional smear-based techniques. However, locally-derived evidence of Xpert MTB/RIF in HIV-infected people is limited. This study evaluates the performance of the Xpert MTB/RIF in HIV-infected patients with smear-negative pulmonary TB (SNTB).

**Methods:**

This was a cross-sectional study in 3 hospitals. The performance of Xpert MTB/RIF was compared with the reference standard of liquid culture and phenotypic drug-susceptibility testing for rifampicin (RIF) resistance.

**Results:**

Out of 123 patients, the median age was 37.0 (IQR: 32.0–41.0) and 81.3% were male. The area under the receiver operating characteristic curve, sensitivity (Se) and specificity (Sp) of Xpert MTB/RIF for pulmonary TB diagnosis were 0.72 (95% confidence interval [CI]: 0.63–0.81), 66.7% (95%CI: 54.8–77.1) and 77.1% (95%CI: 62.7–88.0), respectively, while Se and Sp of Xpert MTB/RIF in detecting RIF resistance were 50.0 (11.8–88.2) and 86.4% (95%CI: 72.7–94.8).

**Conclusion:**

The performance of Xpert MTB/RIF in HIV-infected patients with SNTB for the diagnosis of TB and RIF-resistance was low. Further studies are required to evaluate the results of Xpert MTB/RIF assay in HIV-infected patients with SNTB and the role of Xpert repetition on the same specimens.

## Introduction

Tuberculosis (TB) is the globally leading cause of death among people living with human immunodeficiency virus (HIV), accounting for 1.2 million TB-related mortalities among seronegative persons and 208,000 deaths among HIV-infected patients [1]. The World Health Organization (WHO) estimated that among 10.0 million (range, 9.0–11.1 million) TB cases, the proportion of HIV-infected patients were 8.2% [1]. Additionally, TB remains the leading cause of death among HIV-infected patients, accounting for around one in three AIDS-related deaths [2]. HIV is the most important risk factor for TB infection progressing to TB disease. Therefore, it is necessary to prevent, detect and treat TB in HIV co-infected patients. Nowadays, chest X-ray, acid-fast bacilli (AFB) smear, Xpert MTB/RIF, and culture are the most frequent laboratory methods to detect TB. While culture remains the gold standard for TB diagnosis, Xpert MTB/RIF is recommended as the initial test for TB in HIV-infected patients because its sensitivity is substantially higher than that of AFB smear [3, 4].

Among HIV-infected patients, the clinical manifestations are often atypical, particularly in the late stage of HIV infection, non-cavitary disease, lower lobe infiltrates, hilar lymphadenopathy, and pleural effusion [5]. This results in a low proportion of patients with a positive bacteriological test. In December 2010, The WHO first recommended the use of the Xpert MTB/RIF assay (Cepheid, Sunnyvale, CA, USA). The guidelines for using Xpert MTB/RIF in diagnosing pulmonary and extrapulmonary TB in adults and children were reviewed and updated in 2016 [3, 4, 6, 7]. Xpert MTB/RIF is an automated semiquantitative molecular test that allows for the simultaneous detection of mutations in the rpoB gene. The test integrates three technologies (gene extraction, amplification and recognition) and provides results within two hours. It is a sensitive test for detecting both *M*. *tuberculosis* and RIF resistance, allowing early initiation of appropriate antibiotic therapy. WHO endorses Xpert MTB/RIF as the initial test for presumptive pulmonary TB and MDR-TB. In HIV-infected patients, this test is substantially more sensitive than smear and therefore recommended as an initial test for diagnosing with TB [3, 4].

Xpert MTB/RIF plays a key role in diagnosing HIV-infected patients with negative-smear pulmonary TB (SNTB). This measure can shorten turnaround time and provide additional evidence for TB diagnosis when practitioners wait for culture results. Although the first study of the Xpert MTB/RIF showed a sensitivity ranging between 98% in smear-positive TB and 72% in SNTB, subsequent evidence has shown large variability in SNTB, with sensitivity ranging between 26% and 67% [8–14]. This is particularly common in HIV-infected patients, children or patients with SNTB or extrapulmonary TB [11, 14–16].

Since 2011, the Vietnam National Tuberculosis Programme (NTP) has scaled up Xpert MTB/RIF as an initial test to detect TB and RIF resistance for HIV-infected people [17]. There are several studies about applying Xpert MTB/RIF in the diagnosis of TB among HIV-infected patients [13, 18–20]. However, there is a lack of research on the value of this technique in HIV-infected patients with SNTB. Therefore, this study aims to evaluate the performance of the Xpert MTB/RIF in HIV-infected patients with SNTB.

## Methods

### Study population

This cross-sectional study was conducted from January 2013 to December 2015 in three hospitals in Hanoi, Vietnam, including two hospitals specialized in TB (National Lung Hospital, Hanoi Lung Hospital) and one hospital specialized in HIV (09 Hospital). We consecutively enrolled HIV-infected patients with SNTB ≥ 18 years old. The definition of SNTB was that patients had a chest x-ray and clinical symptoms more than two weeks suggesting pulmonary TB (interpreted by two independent trained doctors) and had at least two expectorate negative AFB smears [21]. The exclusion criteria were patients in severe HIV conditions [22].

### Sample size

This study aims to determine how sensitive Xpert MTB/RIF is in diagnosing HIV-infected patients with SNTB. The proportion of culture-positive TB cases were 68% in HIV-infected patients with SNTB in Vietnam [23]. Previous studies showed that the sensitivity of Xpert MTB/RIF was about 70% [8–14]. The null hypothesis of this study was that the sensitivity of Xpert MTB/RIF was 70%, while the alternative hypothesis was that sensitivity was not equal to 70%. Therefore, the sample size that would need to have 95% confidence and 80% power to detect a difference of 10% from sensitivity of 70% was 123 patients [24].

### Study procedure

All HIV-infected patients with clinically presumptive TB were initially screened for TB by two spontaneous sputum specimens and chest x-ray following national standard practice. Patients who met the inclusion criteria were approached and invited to participate in the study. Patients who consented to participate were enrolled and requested to provide one additional spot sputum sample. When patients could not produce an adequate expectorate, sputum induction was performed by nebulizing sterile 5% saline. This sputum sample was divided into 2 parts for liquid culture and Xpert MTB/RIF. For liquid culture, 5 ml sputum pellets were inoculated on liquid medium (BD BBL Mannula MGIT, Cockeysville, MD, USA) and read using the BD BACTEC Micro MGIT Fluorescence Reader (Cockeysville, MD, USA). The culture-positive samples underwent conventional drug susceptibility testing (DST) for isoniazid (INH), rifampicin (RIF), pyrazinamide (PZA), ethambutol (EMB) and streptomycin (S). The second half of the homogeneous sputum sample was tested with Xpert MTB/RIF by adding the sample reagent to untreated sputum in a 2:1 ratio (1ml of untreated sputum to 2 ml of the sample reagent). After that, 2 ml of this mixture was transferred to the Xpert MTB/RIF cartridge and the cartridge was then loaded into the GeneXpert device. Finally, the results were interpreted and displayed by the GeneXpert system from measured fluorescent signals.

All procedures, including sputum induction, microscopy, Xpert MTB/RIF, liquid culture, and DST, were implemented in each hospital laboratory based on biosafety level III [6]. Sputum specimens from patients were collected in a ventilated sputum collection room and submitted to the laboratory to process within 24 hours [21, 25]. Direct sputum-smear microscopy and Xpert MTB/RIF were performed using good microbiological techniques on an open bench with ventilation [21, 25]. In contrast, procedures related to liquefying specimens for liquid culture and DST were performed in a biological safety cabinet.

We collected data on the DST using the standard conventional drug susceptibility of *M*.*tuberculosis* isolates to INH, RIF, PZA, EMB, and S. Diagnosis of MDR-TB was based on conventional DST method, BACTEC MGIT 960 culture and Xpert MTB/RIF assay. Demographics and chest x-ray results were extracted from patient records.

### Statistical analysis

We provided descriptive statistics on patient demographics and clinical data. We provided medians with interquartile ranges, and conducted the Mann–Whitney U-tests with continuous variables with skewed distribution. Normally distributed continuous variables were presented as mean and standard deviation ranges, and compared by Student’s t-tests. Chi-square or Fisher’s exact tests were employed for dichotomous variables. We calculated AUC-ROC, sensitivity (Se), specificity (Sp), positive predictive value (PPV), negative predictive value (NPV), positive likelihood ratio (PLR), negative likelihood ratio (NLR) with 95% confidence interval (95%CI) based on a binomial distribution. The measure of agreement was used by Cohen’s Kappa statistic. Hypothesis testing was two-sided and p-values less than 0.05 were considered statistically significant. We performed analyses using SPSS 25 (IBM Corp., Armonk, NY, USA).

### Ethics statement

This study was given scientific and ethical approval by the Institutional Ethics and Scientific Review Board of the National Lung Hospital under approval letter number 10/2013/NCKHCS. All patients in this study were informed about the risks and benefits of the study and signed an individual written informed consent.

## Results

From January 2013 to December 2015, we enrolled 123 HIV-infected patients with SNTB, and the majority of patients were male (81.3%). The median participant age was 37.0 years (IQR: 32.0–41.0). At the time of enrolment, 59 (48.0%) patients were on ART treatment, in which 23 (18.7%) patients were on ART treatment for a median of 7.0 (3.0–20.0) months. The CD4 counts were available for 82 (66.7%) patients with a median of 247.0 (100.8–400.0) cells/ml. There were 6 (5.3%) patients on INH prophylaxis before enrolment. Spontaneous sputum specimens were collected in 76 (61.8%) patients and 47 (38.2%) patients required induced sputum collection. Characteristics of patients in this study are shown in Table 1.

**Table 1:** Baseline characteristics of study participants.

Clinical characteristics	All (N = 123)		Spontaneous sputum (N = 76)		Induced sputum (N = 47)		P- value
	N	Value	N	Value	N	Value	
**Age** (years) (median, IQR^a^)	123	37.0 (32.0–41.0)	76	36.0 (31.0–40.0)	47	37.0 (34.0–43.0)	0.254
**Male gender** (%)	100	81.3	65	85.5	35	74.5	0.126
**Body mass index** (median, IQR)	123	17.6 (16.3–29.5)	76	17.9 (16.2–19.6)	47	17.5 (16.0–18.8)	0.358
**Intravenous drug user** (%)	66	53.7	44	57.9	22	46.8	0.231
**Direct contact with individuals with TB**^**b**^ (%)	25	20.3	16	21.1	9	19.1	0.799
**ART**^**c**^ **treatment** (%)	59	48.0	30	39.5	29	61.7	0.016
**Duration of ART among ART experienced patients** (months) (median, IQR)	23	7.0 (3.0–20.0)	10	7.0 (4.3–14.0)	13	7.0 (3.0–25.0)	0.832
**INH**^**d**^ **prophylaxis at any time** (%)	6	5.3	2	2.6	4	8.5	0.201
**CD4 counts at the time of HIV diagnosis** (cells/ml) (median, IQR)	82	247.0 (100.8–400.0)	47	250.0 (114.0–375.0)	35	200.0 (97.0–400.0)	0.409
**Pulmonary cavitation on chest radiograph** (%)	33	26.8	25	32.9	8	17.0	0.054
**Anemia** (%)	97	78.9	57	75.0	40	85.1	0.182

IQR^a^, Interquartile range;

TB^b^, Tuberculosis;

ART^c^, Antiretroviral therapy;

INH^d^, Isoniazid

The results of Xpert MTB/RIF assay against BACTEC MGIT 960 culture are in Table 2. Sixty-one patients (49.6%) were diagnosed with TB by Xpert MTB/RIF, while BACTEC MGIT 960 culture detected TB in 75 patients (61.0%). Diagnostic efficacy of Xpert was assessed in all 123 cases with the overall Se, Sp, PPV and NPV of 66.7% (95%CI: 54.8–77.1), 77.1% (95%CI: 62.7–88.0), 82.0% (95%CI: 72.5–88.7) and 59.7% (95%CI: 50.9–68.0), respectively. Compared with the spontaneous expectorate group, Xpert MTB/RIF in the induced sputum group showed similar results in AUC-ROC and sensitivity tests. However, patients in the induced sputum group had the highest PPV and PLR with 85.0% and 4.58, respectively. The kappa of 0.42 in the whole study and 0.50 in the induced sputum group represents a moderate level of agreement between Xpert and BACTEC MGIT 960 culture.

**Table 2:** Performance of Xpert MTB/RIF assay against BACTEC MGIT 960 culture as the reference standard.

Xpert MTB/RIF^a^	BACTEC MGIT 960 culture								
	All n (%)			Induced sputum n (%)			Spontaneous sputum n (%)		
	Negative	Positive	Total	Negative	Positive	Total	Negative	Positive	Total
**Negative**	37 (30.1)	25 (20.3)	62 (50.4)	18 (38.3)	9 (19.1)	27 (57.4)	19 (25.0)	16 (21.1)	35 (46.1)
**Positive**	11 (8.9)	50 (40.7)	61 (49.6)	3 (6.4)	17 (36.2)	20 (42.6)	8 (10.5)	33 (43.4)	41 (53.9)
**Total**	48 (39.0)	75 (61.0)	123 (100.0)	21 (44.7)	26 (55.3)	47 (100.0)	27 (35.5)	49 (64.5)	76 (100.0)
**Xpert MTB/RIF**	All (95% CI^i^)			Induced sputum (95% CI)			Spontaneous sputum (95% CI)		
**AUC-ROC**^b^	0.72 (0.63–0.81)			0.76 (0.61–0.90)			0.69 (0.56–0.82)		
**Kappa**	0.42 (0.26–0.57)			0.50 (0.26–0.74)			0.35 (0.15–0.56)		
**Se**^**c**^ **%**	66.7 (54.8–77.1)			65.4 (44.3–82.8)			67.4 (52.5–80.0)		
**Sp**^**d**^ **%**	77.1 (62.7–88.0)			85.7 (63.7–97.0)			70.4 (49.8–86.3)		
**PPV**^**e**^ **%**	82.0 (72.5–88.7)			85.0 (65.7–94.4)			80.5 (69.1–88.4)		
**NPV**^**f**^ **%**	59.7 (50.9–68.0)			66.7 (53.4–77.7)			54.3 (42.6–65.5)		
**PLR**^**g**^	2.91 (1.69–5.01)			4.58 (1.55–13.54)			2.27 (1.23–4.20)		
**NLR**^**h**^	0.43 (0.30–0.62)			0.40 (0.23–0.70)			0.46 (0.29–0.74)		

MTB/RIF^a^, Mycobacterium tuberculosis/Rifampicin;

AUC-ROC^b^, Area Under the Receiver Operating Characteristic Curve;

Se^c^, Sensitivity;

Sp^d^, Specificity;

PPV^e^, Positive predictive value;

NPV^f^, Negative predictive value;

PLR^g^, Positive likelihood ratio;

NLR^h^, Negative likelihood ratio;

CI^i^, confidence interval.

There were 75 culture-positive samples in culture, in which 5 cases happened to errors or contaminations while DST was performed. GeneXpert detected 61 positive cases, in which 3 cases did not be distinguished RIF resistance because of errors. Therefore, only 50 cases had both DST and Xpert MTB/RIF results. The results of GeneXpert MTB/RIF assay against standard conventional DST for MDR-TB are also presented in Table 3. Diagnostic efficacy of Xpert for MDR-TB in culture-positive cases was high with overall Sp and NPV of 86.4% (95%CI: 72.7–94.8) and 92.7% (95%CI: 84.9–96.6), respectively (Table 3). However, Se and PPV were less than 50%.

**Table 3:** Performance of Xpert MTB/RIF assay against standard conventional drug susceptibility test for MDR-TB.

GeneXpert MTB/RIF^a^	Drug Susceptibility Test of RIF n (%)		
	Resistant	Sensitive	Total
**Resistant**	3 (6.0)	6 (12.0)	9 (18.0)
**Sensitive**	3 (6.0)	38 (76.0)	41 (82.0)
**Total**	6 (12.0)	44 (88.0)	50 (100.0)
**Xpert MTB/RIF (95% CI**^**i**^**)**			
**AUC-ROC**^b^	0.68 (0.43–0.94)		
**Kappa**	0.30 (0.01–0.58)		
**Se**^**c**^ **%**	50.0 (11.8–88.2)		
**Sp**^**d**^ **%**	86.4 (72.7–94.8)		
**PPV**^**e**^ **%**	33.3 (14.4–59.9)		
**NPV**^**f**^ **%**	92.7 (84.9–96.6)		
**PLR**^**g**^	3.67 (1.23–10.93)		
**NLR**^**h**^	0.58 (0.26–1.30)		

MTB/RIF^a^, Mycobacterium tuberculosis/Rifampicin;

AUC-ROC^b^, Area Under the Receiver Operating Characteristic Curve;

Se^c^, Sensitivity;

Sp^d^, Specificity;

PPV^e^, Positive predictive value;

NPV^f^, Negative predictive value;

PLR^g^, Positive likelihood ratio;

NLR^h^, Negative likelihood ratio;

CI^i^, confidence interval.

## Discussion

Xpert MTB/RIF is a novel PCR-based diagnostic test that is substantially more sensitive for detecting *M*. *tuberculosis* than traditional smear-based techniques. However, there is limited evidence on the value of Xpert MTB/RIF in HIV-infected patients with SNTB. This study showed that the performance of Xpert MTB/RIF in the diagnosis of TB and MDR-TB was fair and induced sputum collection could detect more TB cases than spontaneously expectorated sputum.

In this study, the Xpert MTB/RIF has moderate sensitivity and specificity with 66.7% (95%CI: 54.8–77.1) and 77.1% (95%CI: 62.7–88.0), respectively. A previous study found that the sensitivity of Xpert MTB/RIF in HIV-infected patients with SNTB was 47.3 (95% CI: 29.2–67.0) [14]. HIV co-infection was associated with a significant reduction of NPV and decreasing trend of sensitivity. A multi-center study in the United States, Brazil, and South Africa found that the sensitivity of Xpert MTB/RIF was low in HIV-infected participants with only 52.1% (95% CI: 38.3%–65.5%) [26]. Results in a meta-analysis assessing the accuracy of Xpert MTB/RIF in HIV-positive individuals by smear status also were comparable to our results [13]. Among HIV-infected patients, the pooled sensitivity of Xpert MTB/RIF was 61% (95%CI: 40%-81%) for SNTB compared with 97% (95%: 90%-99%) for smear-positive pulmonary TB [13]. Overall, studies evaluating performance of Xpert MTB/RIF on HIV-infected patients with SNTB had similar research design and concordant results with our study. The moderate sensitivity and specificity could be due to the inability of Xpert MTB/RIF to detect DNA of *M*. *tuberculosis* in concentrations below 131 cfu/ml (the lowest threshold of Xpert MTB can detect TB bacteria), while sputum culture could detect TB bacteria at the threshold of 10–100 cfu/ml [27, 28]. Furthermore, the presence of inhibitors to the gene amplification enzyme (PCR) in the test specimen might present another challenge for the Xpert MTB/RIF [29]. Therefore, the role of Xpert MTB/RIF in diagnosing HIV-infected patients with SNTB should be further optimized as the moderate sensitivity and specificity. These results showed that the final diagnosis should be based on carefully examining clinical symptoms combined with traditional microbiological tests such as sputum smear and culture.

In a previous study on 171 cases of smear-negative and culture-positive sputum, the sensitivity value increased to 72.5%, 85.1%, 90.2% when Xpert MTB/RIF was performed once, twice, and thrice on the same specimen [8]. In Vietnam, Xpert MTB/RIF has been used as the initial test because of the lower sensitivity of AFB smear microscopy and the need for early TB treatment in HIV-infected patients. However, our study showed moderate sensitivity and specificity, so repeating Xpert MTB/RIF test could be a good method to increase its sensitivity. However, the cost of Xpert MTB/RIF is still high compared to smear microscopy, and further studies about the Xpert MTB/RIF performance in HIV-infected patients with SNTB are needed.

The detection of RIF-resistance of Xpert MTB/RIF in this study was lower than in previous studies [10, 30]. However, the sensitivity and specificity of Xpert MTB/RIF for RIF resistant specimens have shown a large variability of 33–100% and 83–100%, respectively [3]. The pooled sensitivity in a meta-analysis was 91% (95%CI: 87–94%); the pooled specificity was 98% (95%CI: 96–99%) [31]. Although this study showed low performance in finding subjects with RIF resistance, it demonstrated an excellent rule-out value for RIF resistance. The discordant results between Xpert MTB/RIF and the conventional gold standard DST had negatively affected treatment decisions in the past [3, 31–34]. Several reasons could explain these differences. Firstly, DST was based on the growth of all *M*. *tuberculosis*, including both drug-resistant and -susceptible strains, while Xpert MTB/RIF detected genetic mutations. Secondly, the MGIT-DST method was imprecise if the mutation was detected on 511Pro, 516Tyr, 533Pro, 572Phe, and several 526 mutations [35]. A number of recent studies found that some strains of *M*. *tuberculosis* with Asp 516Tyr mutation in the rpoB gene could cause low levels of RIF resistance [33, 36]. Therefore, it was still drug-susceptible on MGIT, whereas the Xpert MTB/RIF result would be classified as RIF resistance. These cases would subsequently be identified as false positives. However, Xpert MTB/RIF could also detect silent mutations (nonfunctional gene) and missense mutations (nonfunctional protein) in the rpoB gene, occasionally leading to false-positive RIF resistance [33]. In our study, 3 patients had false-negative results, where RIF sensitive samples yielded resistant isolates on MGIT 960. 2 out of 3 patients were changed to resistance therapy, 1 out of 3 was unable to contact. Causes of false-negative results of Xpert MTB/RIF for RIF resistance have not been well established [37]. Some previous studies showed that although Xpert could detect a larger number of rpoB mutations, it was difficult to find all mutations related to rifampin resistance [38, 39]. A recent study on 370 patients with pulmonary TB mentioned that the presence of wild-type sequences detected by the probes, could be one of reasons leading to false-negative results [40]. The false-positive and false-negative cases in this study were not sequenced to determine the mismatch between the results as well as the likelihood of coinfection. This was a limitation of our study and should be addressed in future research. In order to resolve the discrepancy of Xpert MTB/RIF, culture, and DST in detecting *M*. *tuberculosis* or RIF-resistance, repeat Xpert MTB/RIF, Genotype® MTBDRplus assay, and sequencing of the rpoB gene should be implemented [32]. Additionally, stratifying the clinical risk for TB and drug-resistant TB based on patient characteristics and local epidemiology remains critical to optimizing initial TB treatment.

Our study has several strong points. Firstly, the number of HIV-infected patients meeting the criteria of SNTB was a significant sample size for studying this vulnerable population, which could help to support the accuracy of the results. Secondly, spontaneous sputum and induced sputum were performed to optimize the quality of the sputum samples for testing. Thirdly, Xpert MTB/RIF assay and culture were performed on the identical sample (homogenous sample), which was the best method for evaluating the accuracy of the diagnostic study. However, this study has some methodological limitations due to financial and logistic constraints. The data about history of HIV treatment, including prophylaxis, duration of ART treatment, CD4 counts at the time of HIV diagnosis, was retrospective and declarative, leading to bias. Sequencing was not performed for patients with discordant RIF resistant results between Xpert MBT/RIF and DST, so it was impossible to determine the exact cause for the discrepancy.

## Conclusions

In our study, the diagnostic value of Xpert MTB/RIF in HIV-infected patients with SNTB was moderate compared to BACTEC MGIT 960 and conventional DST. However, Xpert MTB/RIF also showed that it had an excellent rule-out value for RIF resistance. Further studies should be performed to evaluate the performance of Xpert MTB/RIF assay in HIV-infected patients with SNTB and the role of repetition of Xpert MTB/RIF on the same specimens.

## Supporting information

S1 Data (SAV)
